# Venom gland transcriptomes of two elapid snakes (*Bungarus multicinctus *and *Naja atra*) and evolution of toxin genes

**DOI:** 10.1186/1471-2164-12-1

**Published:** 2011-01-03

**Authors:** Yu Jiang, Yan Li, Wenhui Lee, Xun Xu, Yue Zhang, Ruoping Zhao, Yun Zhang, Wen Wang

**Affiliations:** 1 CAS-Max Planck Junior Research Group, State Key Laboratory of Genetic Resources and Evolution, Kunming Institute of Zoology, Chinese Academy of Sciences, Kunming, Yunnan 650223, China; 2 Graduate University of Chinese Academy Sciences, Beijing 100039, China; 3 College of Animal Science and Technology, Sichuan Agricultural University, Sichuan 625014, China; 4 Biotoxin Units, Key Laboratory of Animal Models and Human Disease Mechanisms, Kunming Institute of Zoology, Chinese Academy of Sciences, Kunming, Yunnan 650223, China

## Abstract

**Background:**

Kraits (genus *Bungarus*) and cobras (genus *Naja*) are two representative toxic genera of elapids in the old world. Although they are closely related genera and both of their venoms are very toxic, the compositions of their venoms are very different. To unveil their detailed venoms and their evolutionary patterns, we constructed venom gland cDNA libraries and genomic bacterial artificial chromosome (BAC) libraries for *Bungarus multicinctus *and *Naja atra*, respectively. We sequenced about 1500 cDNA clones for each of the venom cDNA libraries and screened BAC libraries of the two snakes by blot analysis using four kinds of toxin probes; *i.e*., three-finger toxin (3FTx), phospholipase A2 (PLA2), kunitz-type protease inhibitor (Kunitz), and natriuretic peptide (NP).

**Results:**

In total, 1092 valid expressed sequences tags (ESTs) for *B. multicinctus *and 1166 ESTs for *N. atra *were generated. About 70% of these ESTs can be annotated as snake toxin transcripts. 3FTx (64.5%) and *β *bungarotoxin (25.1%) comprise the main toxin classes in *B. multicinctus*, while 3FTx (95.8%) is the dominant toxin in *N. atra*. We also observed several less abundant venom families in *B. multicinctus *and *N. atra*, such as PLA2, C-type lectins, and Kunitz. Peculiarly a cluster of NP precursors with tandem NPs was detected in *B. multicinctus*. A total of 71 positive toxin BAC clones in *B. multicinctus *and *N. atra *were identified using four kinds of toxin probes (3FTx, PLA2, Kunitz, and NP), among which 39 3FTx-postive BACs were sequenced to reveal gene structures of 3FTx toxin genes.

**Conclusions:**

Based on the toxin ESTs and 3FTx gene sequences, the major components of *B. multicinctus *venom transcriptome are neurotoxins, including long chain alpha neurotoxins (*α*-ntx) and the recently originated *β *bungarotoxin, whereas the *N. atra *venom transcriptome mainly contains 3FTxs with cytotoxicity and neurotoxicity (short chain *α*-ntx). The data also revealed that tandem duplications contributed the most to the expansion of toxin multigene families. Analysis of nonsynonymous to synonymous nucleotide substitution rate ratios (*dN*/*dS*) indicates that not only multigene toxin families but also other less abundant toxins might have been under rapid diversifying evolution.

## Background

Snake venoms comprise a diverse array of toxins that have a variety of biochemical and pharmacological functions and can be conveniently classified as hemotoxins and neurotoxins [1]. Recently, it has been documented that most of the snake toxins were recruited or derived from normal body proteins in the common ancestor of venomous squamates (Toxicofera) or advanced snakes (Caenophidia) 100-200 million years ago (mya) [2-5]. Associated with the species radiation of advanced snakes in the Cenozoic era, a predator-prey arms race led to the explosive appearance of toxic arsenals, and typically, several toxin multigene families were generated by gene duplication, followed by fast diversification [4,6]. The birth-and-death model was proposed to explain the emergence of taxon-specific toxin groups [7]; *i.e*., new toxin genes consistently emerged through gene duplication with the divergence of taxa, but some toxin genes got deleted or were degenerated in other lineages. However, due to the low-depth sequencing of toxin transcripts for each species, fast evolution of toxin genes, and lack of genome sequences, the detailed elaboration of snake venom evolution is still unclear.

The Elapidae family (elapids), which has approximately 300 venomous snakes in 61 genera, is a monophyletic group among advanced snakes [8,9]. Several broad radiated lineages (diverged rapidly between around 31 and 26 mya, based on fossil records and molecular evidence [9]) have been identified within the Elapidae, including the Afro-Asian cobras, Oriental kraits, Asian-American corals, and Australian and marine snakes, which are well known to be the most toxic snakes in the world. So far, the gene expression profiles of venom glands from four species [10-12] have been reported using EST sequencing. However, the kraits (genus *Bungarus*) and cobras (genus *Naja*), as the most diverse and representative toxic elapids in the old world [7], lack genomic and venom EST data.

In the present study, we prepared cDNA libraries from the venom glands of the two representative old world elapid snakes, *Bungarus multicinctus *and *Naja atra*, and sequenced about 1500 clones for each library. We also constructed genomic bacterial artificial chromosome (BAC) libraries for the two snakes and conducted a screen for venom genes. Our results identified many new snake toxins, such as multiple groups of 3FTxs, some novel lectins, and a peculiar natriuretic peptide (NP), and revealed that toxin genes have experienced rapid evolution and gene expansion.

## Results and Discussion

### Venom gland cDNA libraries and ESTs

We constructed two cDNA libraries from the venom gland tissues of *B. multicinctus *and *N. atra*, respectively, and randomly selected 1441 and 1534 clones for sequencing. From these clones, we successfully obtained 1092 expressed sequences tags (ESTs) for *B. multicinctus *and 1166 ESTs for *N. atra*. Using CAP3 software with a cutoff of 95% identity [13], the *B. multicinctus *ESTs were grouped into 294 clusters (including 83 contigs assembled by two or more ESTs and 211 singlets), whereas the *N. atra *ESTs were grouped into 339 clusters (including 62 contigs and 277 singlets). Using BLAST for functional annotation, complete lists with putative gene identifications for these EST clusters are provided in Additional file 1. Most ESTs could be matched to reported snake toxins (69.51% in *B. multicinctus *and 70.24% in *N. atra*). The toxin clusters showed notably high redundancy (14.32 ESTs/cluster in *B. multicinctus*, 12.41 ESTs/cluster in *N. atra*), in contrast to the low redundancy of non-toxin clusters (1.40 ESTs/cluster in *B. multicinctus*, 1.31 ESTs/cluster in *N. atra*) (Table 1), indicating that the toxin genes were highly expressed.

**Table 1:** Composition of ESTs obtained from B. multicinctus and N. atra venom glands

	Transcripts category	Number of ESTs	Number of clusters	Redundancy (clones/clusters)	Representation over total clones (%)
*B. multicinctus*	Toxins	760	54	14.07	69.60
	Non-toxins	288	205	1.40	26.37
	No hit	44	35	1.26	4.03

*N. atra*	Toxins	819	66	12.41	70.24
	Non-toxins	304	232	1.31	26.07
	No hit	43	41	1.05	3.69

**Figure 1:**
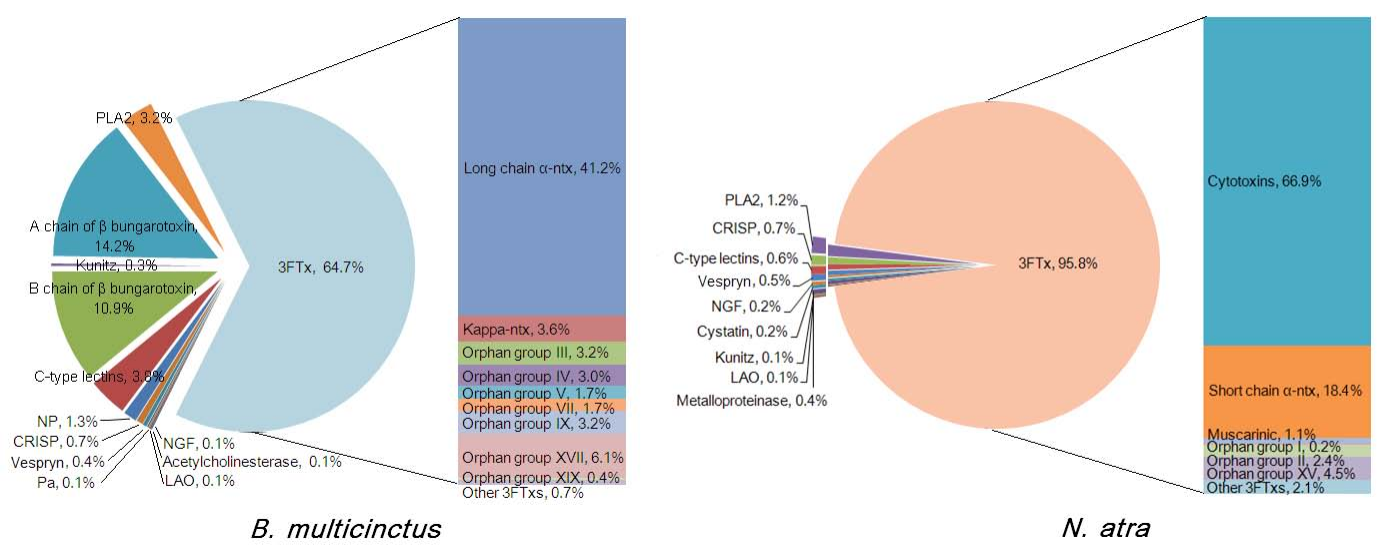
**Distribution of toxins in the venom gland transcriptomes of *B. multicinctus *and *N. atra***.

In this study, we focused on the toxin ESTs; i.e., 26 contigs and 27 singlets in *B. multicinctus*, and 18 contigs and 48 singlets in *N. atra *(Figure 1). Except the dominant 3FTx, we also observed eight less relatively abundant venom families both in *B. multicinctus *and *N. atra*: phospholipase A2 (PLA2), C-type lectins, kunitz-type protease inhibitor (Kunitz), L-Amino oxidase (LAO), nerve growth factor (NGF), cysteine-rich secretory protein (CRSIP), and vespryn. However, NP, presynaptic (*β*) bungarotoxin, prothrombin activator (Pa), and acetylcholinesterase were only detected in *B. multicinctus*, while metalloproteinase and cystatin were only found in *N. atra*.

In snake venoms, 3FTx, PLA2, Kunitz, C-type lectins, and metalloproteinase have been reported to be the major multigene toxin families [6], and they comprised 97.1% in *B. multicinctus *and 98.0% in *N. atra *in our toxin EST data. We also identified a peculiar cluster of NP precursors with tandem NPs. Besides, *β *bungarotoxin is a Bungarous-specific toxin, which is a heterodimeric protein complex of phospholipase A2 (A chain) and kunitz peptides (B chain) covalently linked by one disulphide bridge [4,14]. Here, we detected 7 clusters (108 ESTs) of A chain of *β *bungarotoxin and 4 clusters (83 ESTs) of B chain as a major toxin in *B. multicinctus *(25.1% of all toxin ESTs).

### Analysis of toxin genes

#### Three-finger toxins (3FTxs)

3FTxs are neurotoxins and can bind to very different neurological targets (neuronal, pre-synaptic, postsynaptic, and synaptic); some of them also have non-neurotoxic activities (cytotoxicity and platelet inhibition) [4,5]. Its family members have a similar structure, with three loops and four conserved disulfide bridges, and contain about 60-74 amino acid residues. The ancestral 3FTx was thought to have 10 cysteines and acquired some amelioration: deletion of the ancestral C^2 ^and C^3 ^cysteines potentiated alpha-neurotoxic activity and acquired additional newly evolved cysteines [7]. In 2003, Fry grouped 33 types of 3FTxs from 276 protein sequences, with a number of "orphan groups" of 3FTxs whose functional roles are not yet known [7]. Since then, many other kinds of 3FTxs have been successively detected [12,15,16]. So far, more than five hundred variant 3FTx sequences across the entire advanced snakes have been observed. A birth-and-death mode [7] and a mechanism of duplication and divergence [17], allowing venom snakes to adapt to a variety of prey, were proposed to explain the generation of the 3ftx superfamily. But, the evolutionary relationships of all kinds of 3FTxs are still uncertain, such as the notable alpha neurotoxic clades (short chain, long chain, kappa, and type III), which are not monophyletic, based on the phylogenetic tree of 3FTxs constructed by Fry [4]. It is in contrast to previous hypotheses that alpha neurotoxins (*α*-ntx) diverged from a common ancestral gene that separated before the divergence of the elapid sub-family [18].

**Figure 2:**
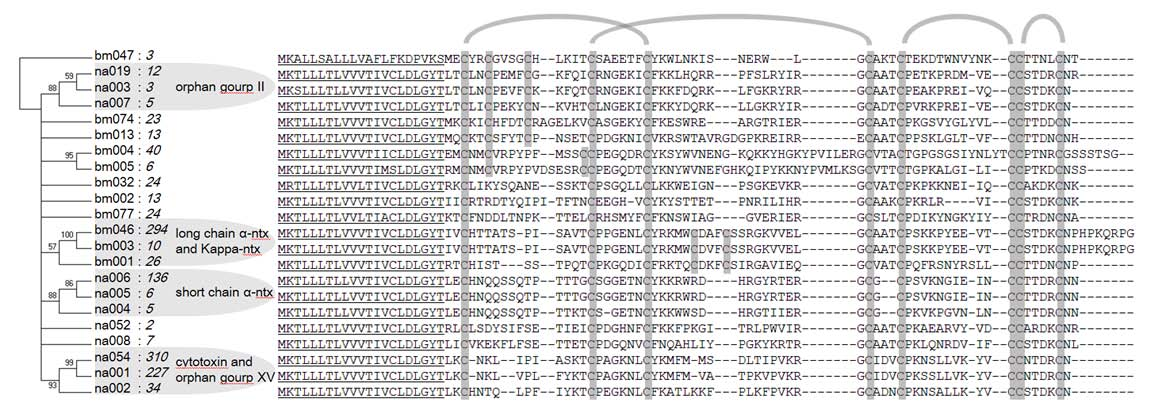
**Representative alignments of translated 3FTx contigs from *B. multicinctus *and *N. atra***. Sequences can be organized in different groups, as revealed by a neighbor-joining tree on the left. Only branches with 50% or greater bootstrap support are shown. The number of ESTs is shown in italics, and the predicted signal peptide is underlined.

The analysis of the *B. multicinctus *3FTxs revealed that 491 clones (64.5% of all toxin ESTs) could be grouped into 12 contigs and 9 singlets, and a total of 785 *N. atra *clones (95.8% of all toxin ESTs) fell into 12 contigs and 35 singlets. We notice that all the contig sequences encoded full-length proteins, except two contigs (na009 with 3 clones, bm006 with 7 clones), which are lacking exon2, probably resulting from alternative splicing. Since 3FTxs are the dominant toxins in both *B. multicinctus *and *N. atra *and show a greater diversity of family members, we classified them basically based on the number of cysteines following the nomenclature of Fry [7] (Figure 2 and Additional file 1). Divergence analysis shows that four of the 3FTx groups had relatively low intra-divergence levels. Three of the groups--long chain *α*-ntx (also together with kappa-ntx, 69.2% of total 3FTx ESTs in *B. multicinctus*), cytotoxin (also together with orphan XV group, 74.5% of total 3FTx ESTs in *N. atra*), and short chain *α*-ntx (19.2% of total 3FTx ESTs in *N. atra*)--were much more abundant than the rest of the 3FTxs (Figure 1). The other group, orphan gourp II (2.5% of total 3FTx ESTs in *B. multicinctus*), have 10 cysteines and thus could be considered as an ancestral-like 3FTx group.

If the nonsynonymous to synonymous substitution rate (*dN*/*dS*) for one protein-coding sequence is significantly higher than 1, it indicates a trend toward accelerated evolution. Because short coding sequences of 3FTxs have high variation (fast adaptive evolution) with multiple indels or even frameshifts between different 3FTx toxin groups, this maybe led to inaccurate multiple alignments and ambiguous phylogenetic relationships. So, we only computed *dN*/*dS *rates within each of the four major 3FTx groups using PAML [19]. We observed that many *dN*/*dS *values are large than 1 (Table 2). As an extreme example, we noticed that the two dominating 3FTx contigs (na001 with 227 ESTs, na054 with 310 ESTs) occupied 65.6% of all toxin ESTs in *N. atra*, and all of the 15 differences between these two cytotoxin contigs were non-synonymous substitutions, which is significantly different from neutral expectation (*p *= 0.008, one-tail test) by Fisher's Exact Test [20], suggesting a recently accelerated diversification in this major group of 3FTxs in *N. atra*.

**Table 2:** Likelihood ratio test of positive selection in toxins

gene group	n	Lc	S	M0	M2a model ω (*p_1_*)	M8 model ω (*p_1_*)	M1a-M2a 2ΔlnL	M7-M8 2ΔlnL
				model				
orphan II	4	67	0.97	1.06	6.56 (0.102)	6.57(0.102)	4.98	2.54
long chain	4	72	2.43	2.41	8.56 (0.547)	8.55(0.551)	38.98**	19.47**
short chain	4	64	1.09	1.37	5.69 (0.309)	4.21(0.461)	7.77*	5.81
cytotoxin	4	64	1.53	2.66	19.5 (0.505)	19.5(0.505)	26.52**	17.25**
PLA2 (only)	4	125	1.66	0.82	3.16 (0.362)	3.01(0.391)	13.78**	6.91*
PLA2	6	125	2.43	0.95	4.40 (0.300)	4.34(0.306)	30.24**	15.74**
lectins	7	134	3.67	1.25	3.36 (0.341)	3.32(0.352)	27.26**	14.30**
Bbeta	4	61	1.73	0.80	10.8 (0.200)	10.8(0.200)	25.18**	12.73**
Kunitz	3	58	0.87	2.49	19.7 (0.312)	18.3(0.318)	18.98**	11.90**
LAO	5	493	0.60	1.18	3.67 (0.294)	3.67(0.293)	29.13**	14.58**
Metal	8	605	2.52	1.06	4.30 (0.227)	4.23(0.238)	179.63*	100.69**
CRISP	8	221	1.46	1.19	3.69 (0.308)	3.70(0.308)	36.62**	18.39**
NP	6	50	5.17	0.48	10.6 (0.122)	9.76(0.122)	11.91**	5.54
NGF	9	117	0.94	1.41	8.08 (0.151)	7.84(0.154)	39.94**	19.96**
vespryn	7	159	0.30	0.87	3.61 (0.273)	3.61(0.273)	6.66*	3.34

Note: *n *represents number of sequences in a gene group. *L*c represents number of codons in the gene after alignment gaps are removed. *S *is the tree length, measured asqqqq the number of nucleotide substitutions per codon. ω is the estimate of the dN/dS ratio under the M0, M2a or M8 model [19]. The proportions of sites under positive selection (*p_1_*) are given under the M2a or M8 model. The 2ΔlnL parameter in the tests of M1a versus M2a or M8 versus M7, indicating positive selection is marked with *. *: significant at 5% level; **: significant at 1% level. PLA2 (only) = Phospholipase A2, Bbeta = B chain of *β *bungarotoxin, Kunitz = Kunitz-type protease inhibitor, Metal = Metalloproteinase.

We also noticed a special contig, bm047 (a homologous protein previously isolated in *Bungarus candidus *by Kuhn *et al*. [21], named bucandin), which was conspicuously different from other contigs and even had a unique signal peptide (Figure 2). Based on the phylogenetic analysis, contig bm047 might have diverged from other 3FTx genes at an early stage of the origin of snakes (data not shown). It is possible that bm047 has a conserved function and has not diversified into a multi-copy gene family.

#### Phospholipase A2 and A chain of *β *bungarotoxin (PLA2)

Elapidae venom PLA2 exerts a multiplicity of novel, nonenzymatic activities, including neurotoxic and antiplatelet activity (group I), whereas viperid venom PLA2 is synovial-type (group II) PLA2 [22]. The elapid PLA2, which has 120 amino acids and 6 or 7 disulfide bonds, was thought to be derived independently from nontoxic pancreatic-type PLA2 [23].

We identified four clusters of PLA2-like ESTs with intact coding sequence (CDS) in *B. multicinctus *and one full-length cluster in *N. atra*, which covered the major PLA2 types detected before in *B. multicinctus *and *N. atra*. In *B. multicinctus*, one cluster (bm008) enclosing 21 ESTs showed 100% identity with the PLA2 sequence of *B. multicinctus *in GenBank (X53406.1). Another three clusters in *B. multicinctus *were similar to the A chain of *β *bungarotoxins, with a notable substitution at residue 99 (from Leu to Cyc) compared with cluster bm008, which may be important for the formation of interchain disulfides in *β *bungarotoxin. Based on the gene tree, it is clear that A chain of *β *bungarotoxin in *B. multicinctus *quite possibly duplicated from the PLA2 gene in *B. multicinctus*. We estimated an average *dS *value of 0.27 between PLA2 contig bm008 and the A chain of *β *bungarotoxins, which is smaller than the *dS *value (0.29) between bm008 and the *N. atra *PLA2 na022, indicating that the A chain of *β *bungarotoxin might have derived from PLA2 after the divergence of *Bungarus *and *Naja*.

#### Kunitz-type protease inhibitor and B chain of *β *bungarotoxin (Kunitz)

Kunitz belongs to the superfamily of bovine pancreatic trypsin-like inhibitors (BPTIs), with the ancestral function of inhibiting a diverse array of serine proteases and with a peptide chain of around 60 amino acid residues and 3 disulfide bonds [3,24]. It evolved the toxin ability to inhibit various physiological processes, such as blood coagulation, fibrinolysis, host defense, and action potential transduction. In elapid venoms, besides the common Kunitz with trypsin or chymotrypsin inhibitor activity, the dendrotoxins in *Dendroaspis *have presynaptic neurotoxicity [25], and the B chain of *β *bungarotoxin is thought to act as an affinity probe to guide the A chain to its target [14].

In *B. multicinctus*, four clusters (83 ESTs) of the B chain of a *β *bungarotoxin-like sequence and one partial kunitz-type protease inhibitor-like cluster (2 ESTs) were obtained. In *N. atra*, only one singlet of a kunitz-type protease inhibitor-like EST was detected. To understand the evolutionary relationship of the kunitz-type protease inhibitor and B chain of *β *bungarotoxin, we constructed a phylogenetic tree (Additional file 2: Figure S1). We also obtained an average ~0.3 *dS *value between the kunitz-type protease inhibitor and B chain of *β *bungarotoxin. Compared with the similar *dS *value of 0.27 between PLA2 contig bm008 and A chain of *β *bungarotoxin, we propose that the A chain and B chain of *β *bungarotoxin evolved separately from PLA2 and Kuntiz around the same time as the common ancestor of *Bungarus*.

#### C-type lectin

C-type lectins or C-type lectin-like proteins are ubiquitous components of animal venoms and contain distinct diversified subgroups, including true lectins, coagulant proteins, platelet aggregation agonists, and platelet aggregation antagonists [26]. The elapid C-type lectins are arranged inside the true lectins group, except one *M. corallinus *C-type lectin [12]. The true lectins cause aggregation of erythrocytes in a dose-dependent manner, which are normally composed of two covalently linked identical subunits, each consisting of 135-141 amino acids [26].

Here, 29 C-type lectins-like ESTs were grouped into 9 clusters (4 contigs and 5 singlets) in *B. multicinctus*, while only one contig from 5 ESTs was identified in *N. atra*. Interestingly, all of these clones do not match the previously sequenced C-type lectins in *B. multicinctus *and *N. atra*. Phylogenetic analysis revealed that a cluster of three contigs (bm016, bm017, bm018) that we sequenced were grouped separately from all of the known *Bungarus *lectins, indicating that this is a novel cluster that diverged from the main group (Additional file 2: Figure S2).

#### Metalloproteinase

Metalloproteinase is the dominant component in viperid venoms, which exerts anticoagulant or coagulant effects and results in a severe bleeding by interfering with blood coagulation and hemostatic plug formation or by degrading the basement membrane or extracellular matrix components of the victims [27]. But, few metalloproteinases are detected in elapids [28], which produce long cDNA products (about 1.8 kb). Here, three partial singlet ESTs in *N. atra *represented two kinds of metalloproteinase isoforms, which were respectively similar to EF080840 in *N. atra *and AY101383 in *Naja mossambica *as P-III type metalloproteinases.

#### Natriuretic peptide (NP)

The NP family functions to control natriuresis, diuresis, blood pressure, homeostasis, and inhibition of aldosterone secretion in all vertebrates or is used by snakes to interrupt these physiological processes of preys [29,30] and thus has attracted attention as ideal candidates of hypotensive and vasodilator agents. It has a conserved ring structure, consisting of 17 amino acids, with extensions of a few amino acids in the two termini. Four major members of the NP family have been indentified in vertebrates--atrial NP (ANP), B-type NP (BNP), C-type NP (CNP), and ventricular NP (VNP)--and structurally, CNP lacks the C-terminal tail, whereas other NPs have both tails [29,31]. All of the snake venom NPs seem to have originated from a common ancestral venom, CNP, and a complete snake CNP pro-peptide is constituted by a signal peptide, a linker, a flexible N-terminal extension (also named the CNP-53 isoform, with a 17-aa core ring), and a mature peptide (CNP-22). Nevertheless, snake NPs have recruited some new segments, such as several bradykinin-potentiating peptides (BPPs), located in the NP precursor in the Viperidae family, whereas an alterable C-terminal extension was acquired in the Elapidae family, which may result in novel physiological actions [13,32,33].

**Figure 3:**
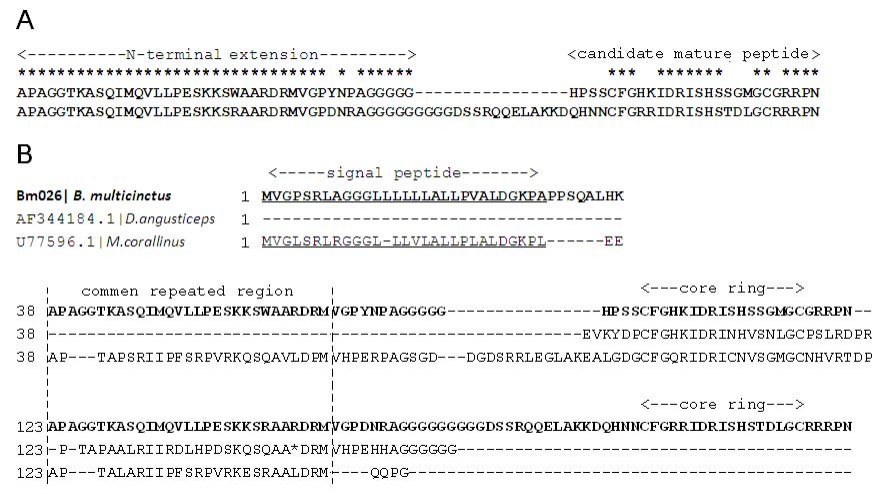
**Alignments of natriuretic peptide (NP) sequences**. (A) Alignment between putative peptides of contig bm026 (38-104) and bm026 (105-187), and the conserved amino acids are indicated by *. The predicted regions of the N-terminal extension and mature natriuretic peptide are indicated above the alignment. (B) Bm026, the NP precursor in *B. multicinctus *(bold), is aligned to other elapid NP sequences. A repeated region is shown between dashed bars, and the predicted regions of signal peptide and core ring are indicated above the alignment.

Here, we detected one contig (bm026 with 10 ESTs) only in *B. multicinctus*, and this transcript had a region containing two tandem CNP-53 repeats. We noticed that this pair of tandem duplicates had different putative mature peptides (10 differences in a 69-bp sequence) but had almost identical N-terminal extensions (3 differences in a 123-bp sequence) (Figure 3A), with statistically significant region-biased mutations (*p *= 0.006, two-tail test) by Fisher's Exact Test, which may represent a functional differentiation between the two NPs. Two possible evolutionary scenarios can explain this pattern: a) this is an old duplicate event, but the N-terminal extensions have a recent conversion or may be under strong functional constraint; and b) this is a young duplicate event, and the putative mature peptides evolved very fast. However, based on the alignment of NP sequences in Elapidae venoms (Figure 3B), we noticed that bm026 and another unusual NP (U77596 from *M.corallinus*) had a common repeated region [12,34]. We also found that the closest sequence of bm026 was the DNP in green mamba snake (*Dendroaspis angusticeps*), which has attracted attention abroad for its novel long C-terminal extension [35], and Figure 3B shows that the C-terminal extension of DNP is homologous with the common repeated region. Based on these clues, we propose that the duplicate event may have occurred in the ancestor of *Bungarus*, *Dendroaspis*, and *Micrurus*.

#### Other toxins

In the cDNA libraries, except for the major multigene toxin families, there were some other toxins that were widely found in snake venoms but that only constituted a small part of the total ESTs in a species. Most of these toxins had been recruited into the venom system before the last common ancestor of elapid and viperid snakes with assistant toxic functions or unknown functions, such as CRISP, NGF, LAO, cystatin, and acetylcholinesterase [4,5]. Besides, vespryn (also named Ohanin, as first detected in *Ophiophagus hannah*) and prothrombin activator (similar to the mammalian blood coagulation factor Xa) were only found in that elapid species so far [36].

In this study, we obtained many full or partial novel sequences of these less abundant toxins: one cluster in *B. multicinctus *and three clusters in *N. atra *coding for CRISP, one cluster in *B. multicinctus *and one cluster in *N. atra *coding for NGF, one cluster in *B. multicinctus *and one cluster in *N. atra *coding for LAO, three clusters in *B. multicinctus *and one cluster in *N. atra *coding for vespryn, one cluster coding for cystatin, and one cluster coding for acetylcholinesterase in *N. atra*.

#### Comparison of the major venom components among elapid snakes

Previously several other transcriptomes of the elapid venom gland had been reported, such as marine snakes (*Lapemis curtus *and *Acalyptophis peronii*), Australian snake (*Austrelaps labialis*), and coral snake (*Micrurus corallines*) [10-12]. However, besides 3FTxs, PLA2 is also another major venom component in these species. We also noticed that the major types, 3FTxs, are very different. The major 3FTx types from the marine and Australian snakes are long chain *α*-ntx and short chain *α*-ntx, whereas *Micrurus corallines *mainly contains orphan gourp XII and some unknown groups of 3FTxs. In contrast, the major components of *B. multicinctus *venom transcriptome are 3FTxs (mainly belong to long chain *α*-ntx group) and recently originated *β *bungarotoxin while the *N. atra *venom transcriptome mainly contains cytotoxin and short chain *α*-ntx groups of 3FTx. Other less abundant toxins were not observed in the transcriptomes of the marine and Australian snakes, maybe due to the small number of sequenced clones.

Besides transcripts, some studies also described peptide sequences for some elapid snake toxins. A study characterizing venom proteins of *N. atra *identified 124 protein segments or peptides [37], 74% of which belonged to 3FTxs and 11% were PLA2. In another study of *Naja kaouthia *venom proteomics [38], 61 venom proteins segments or peptides were identified, most of which were covered by our EST sequence, except the cobra venom factors. Two studies on coral snakes identified 26 and 11 toxin proteins or peptides, respectively [39,40], 3FTx and PLA2 proteins were also the major toxin components. So, the major toxins are basically consistent with our results observed in the *N. atra *venom gland transcriptome while *Bungarus *has quite different components. However, it seems the PLA2 transcripts have higher expression efficiency, considering that the PLA2 ESTs only comprise 1.2% of our toxin ESTs in *N. atra *while PLA2 peptides were observed to compose of a considerable amount in the *N. atra *venom [37].

#### Analyses on the ratios of nonsynonymous to synonymous substitution rates (*dN*/*dS*) for the protein-coding ESTs of all toxin genes

It has been known that the 3FTx and PLA2 toxin multigene families have been subjected to positive Darwinian selection [6,41], but whether this has happened in the less abundant toxins is still unknown. We used the maximum likelihood model to estimate the *dN*/*dS *ratios of all kinds of toxins in elapid snake venoms. The representative sequences used here were from our EST data or downloaded from the GenBank database (Additional file 2: Table S1). Except for orphan group II of 3FTxs in *B. multicinctus*, almost all of the toxins had some sites that showed evidence of positive selection dN/dS ratios, and the dN/dS ratios for these sites ranged from 3.01 to 19.52 (Table 2). These results suggest that most toxin genes in elapid venoms might have been subjected to strong pressure of prey species shifts or arsenal competition, leading to the accelerated innovation of antigenic epitopes in toxins.

### BAC Library construction and sequencing of 3FTx genes

In order to understand the gene structure of toxin genes, two independent BAC libraries containing 73,728 (*B. multicinctus*) and 82,944 (*N. atra*) clones, with average inserted genomic segments of 120 and 100 kb respectively, were constructed and arrayed into 384-well microtiter plates. Examples of 16 digested clones are presented in Additional file 2: Figure S3. We estimated that the percentage of clones without inserts was about 10%. Based on the snake's haploid genome size of 2.38 pg for *Bungarus fasciatus *and 2.59 pg for *Naja haje *[42], we estimated the genome coverage of the libraries to be around 3.3 × for *B. multicinctus *and 2.9 × for *N. atra*. These coverages theoretically provide 97.6% and 96.0% probability of obtaining any unique sequence in the *B. multicinctus *and *N. atra *BAC libraries, respectively, assuming random cloning [43].

We used 3FTx, PLA2, Kunitz, or NP toxin probes to screen the two BAC libraries and identified 50 positive BAC clones for *B. multicinctus *and 21 for *N. atra*. The screening results are listed in Table 3. Unfortunately, we did not find positive PLA2 clones for *N. atra*. In addition, we identified positive NP BAC clones in *N. atra*, but we did not observe ESTs from its cDNA library.

**Table 3:** Number of positive toxin BAC clones identified by different toxin probes

	number of positive toxin BAC clones		probe types used in blot screening	
	***B. multicinctus***	***N. atra***	***B. multicinctus***	***N. atra***

3FTx	23	16	3FTx long chain *α*-ntx	3FTx short chain *α*-ntx
			3FTx kappa-ntx	3FTx cytotoxin
			3FTx orphan group IV	3FTx orphan group I
			3FTx orphan group IXX	3FTx orphan group II
PLA2	16	0	*β *bungarotoxin A-chain	PLA2
Kuntiz	10	5	*β *bungarotoxin B-chain	
NP	3	1	NP	

Sum (remove overlap clones)	50	21	Totaled with 7 probes	Totaled with 5 probes

**Figure 4:**
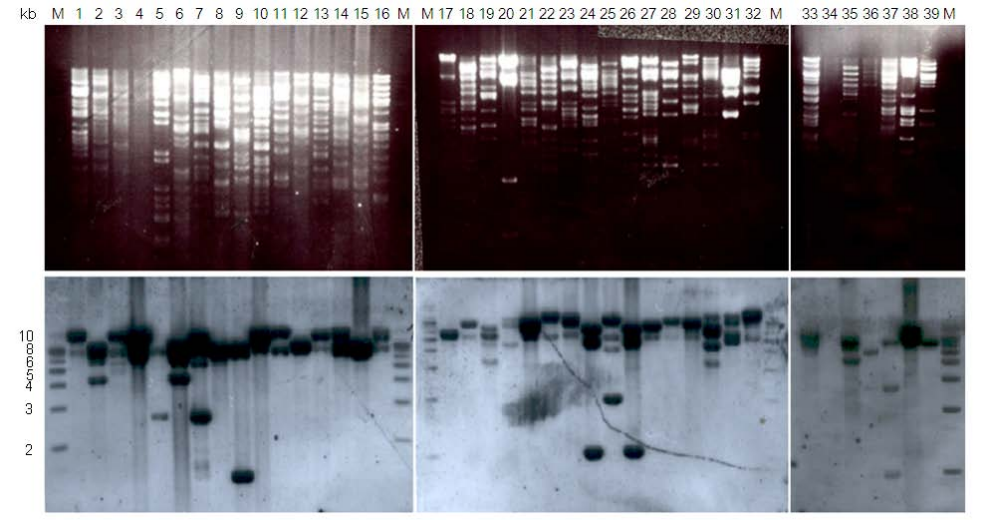
**Blotting analysis of 39 3FTx probe-positive BAC clones**. 3FTx probe-positive BAC clones were digested with *Eco*R I and analyzed on 0.8% agarose gels prior to blotting on a nylon membrane (upper panel). The 3FTx gene-containing fragments were identified by hybridization with digoxigenin-labeled 3FTx probes (lower panel). The positive bands were used as a control to cut and purify 3FTx gene-containing fragments in a second gel. Lanes: M, 1 kb DNA ladder; 1-39, 3FTx probe-positive BAC clones (Additional file 2: Table S2).

In order to understand evolution patterns in the 3FTx super-family, we tried to subclone and sequence the 3FTx toxin genes. There were 39 BAC clones showing 3FTx-probe positivity in this study. As the major toxin in elapid venoms, the structure and organization of 3FTx toxin genes have clearly been found to consist of three exons and two introns in a region of about 2.5 kb [18]. Based on the results of *Eco*R I digestion and agarose gel electrophoresis separation, most of the 39 BAC clones had multiple 3FTx toxin gene fragments, whose sizes were from 5 kb to 10 kb (Figure 4). Considering that the 3FTx toxin genes are about 2.5 kb long and seldom contain *Eco*R I sites, these multiple 3FTx toxin gene fragments, located in the same BAC clone, are very likely to be tandem duplicates.

Excluding some redundant BAC clones with the same 3FTx toxin gene restriction fragments, we obtained 25 RFLP-unique 3FTx toxin BAC clones, indicating there are at least 13 and 12 segmental duplicate areas of 3FTx toxin genes, respectively, in *B. multicinctus *and *N. atra*. Because of the existence of tandem duplicates, it is hard to sequence these clones. Finally, we got 18 and 22 complete or partial 3FTx toxin gene sequences by constructing subcloning libraries and genomic walking, respectively, for *B. multicinctus *and *N. atra*. The 3FTx toxin gene sequences that were derived from the same BAC clone were defined as putative tandem duplicates. There are 5 putative tandem duplicates in *B. multicinctus *and 7 in *N. atra*. The nucleotide substitution rate per site (*Kn*) in the non-coding regions (intron I and intron II of 3FTx gene) between the tandem duplicates is shown in Table 4. We noticed that most of the potential tandem duplicates had low *Kn *values (<0.05), indicating that most duplication events happened recently. It also suggests that fast 3FTx gene expansion is still ongoing in the two elapids.

**Table 4:** Pairwise genetic distance among putative tandem duplicates

BAC name	tandem duplicates	Average pairwise distance	
		
	number	Intron I	Intron II
N044_7B	4	0.018	0.029
N072_20 m	3	0.058	0.012
N036_12b	2	0.018	0.000
N052_20J	2	0.007	0.002
N076_21 m	2	0.128	0.014
N061_6e	2	0.030	0.002
N031_7f	2	0.011	0.002
B218_4L	2	0.004	0.002
B254_9H	2	0.010	0.007
B241_M14	2	0.002	0.009
B244_1O	2	0.154	0.005
B132_1G	2	---	0.103

**Figure 5:**
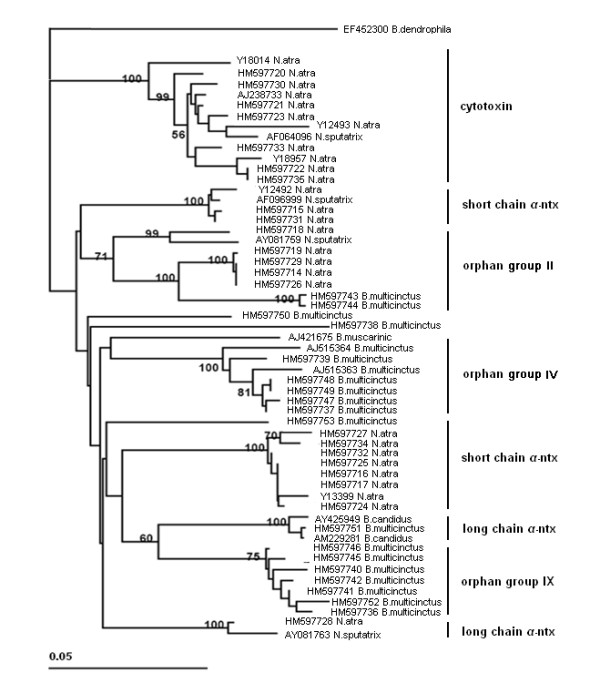
**Phylogenetic tree constructed using 3FTx intron II sequences with the maximum likelihood method in phylip**. The intron sequences were from our BAC libraries and the GenBank database, both in the genus of *Bungarus *and *Naja*, using a colubrid (*Boiga dendrophila*) 3FTx as the outgroup. Numbers above branches indicate posterior probability values higher than 50%. Sequence names are labeled according the GenBank Accn. Number, followed by the species they were identified from. The sequences are classified basically based on the CDS, following the nomenclature of Fry [7].

Based on the best Blast hits to the GenBank database and our EST data, all of the 3FTx toxin genes were classified and annotated (Additional file 2: Table S2). About 65% (26/40) of the gene sequences showed lower than 95% identity with 3FTx toxin gene sequences from the GenBank database, indicating that most of the 3FTx toxin genes that we sequenced are novel sequences (Figure 5). Parts of the 3FTx genes that we sequenced, especially a big group that looked similar to orphan group II, could not match any ESTs from the two cDNA libraries, indicating that a large number of lower-expression 3FTx toxin genes were not detected in previous studies.

Because the coding sequences of 3FTx genes are short and too diversified, it is difficult to construct an accurate phylogenetic relationship for all these diverse subgroups of 3FTx. So, we tried to use only the intron sequences for genic phylogenetic analyses, especially using intron II, which has a stable length of about 550 bp [44]. But, the 3FTx gene tree (Figure 5) still confuses us with multiple radiated sub-families, just like the phylogenetic relationship of the 3FTx constructed by Fry [4,7]. Taking *α*-ntx for example, we observed two monophyletic lineages of short chain *α*-ntx from *N. atra *and two monophyletic lineages of long chain *α*-ntx, respectively, from *B. multicinctus *and *N. atra*, which indicates that multiple groups of *α*-ntx separated before the divergence of *Bungarus *and *Naja*. Considering that the long chain *α*-ntx originated mainly by one splicing site shifting event from short chain *α*-ntx [18] and that both the short and long chain *α*-ntx coexist in some Australian and marine snakes, such as *Austrelaps labialis *[11], *Lapemis curtus *[10], and *Laticauda semifasciata *[18], the hypotheses that *α*-ntx diverged from a common ancestor seems reasonable. So, it is very likely that these broad radiated groups of 3FTx (containing the multiple *α*-ntx, cytotoxin, and other orphan 3FTx groups) appeared right before the explosive speciation of major elapid subfamilies in the mid-Oligocene [9]. In brief, our data suggest that 3FTx genes probably experienced family explosion and thereby generated the major groups before the species radiation of Elapidae subfamilies. After that, by massive duplication, perhaps mainly by tandem duplication, the 3FTx gene family acquired numerous new members and became the most abundant toxin family in the two elapid venoms.

## Conclusions

Both *B. multicinctus *and *N. atra *venoms are very toxic, respectively, with LD50 of 0.108 mg/kg and 0.29 mg/kg when injected subcutaneously (Venomdoc database from Fry), and they belong to two closely related genera [9]. However, the major components of the *B. multicinctus *venom transcriptome are neurotoxins, including long chain *α*-ntx and *β *bungarotoxin, whereas the *N. atra *venom transcriptome mainly has cytotoxicity and a little bit of neurotoxicity from short chain *α*-ntx. In this study, we present the toxin profiles of *B. multicinctus *and *N. atra *by sequencing their venom gland transcriptomes. Then, the BAC libraries for these two elapids were constructed, and 25 BACs containing 3FTx genes were partially sequenced. The data revealed that tandem duplications contributed the majority of the expansions of toxin multigene families in the two elapids. We detected positive selection in every toxin subfamily and found that not only the multigene toxin families but also the less abundant toxins were under rapid adaptive evolution.

## Methods

### Materials

Fresh venom glands and blood cells were obtained, respectively, from an individual of *Bungarus multicinctus *and *Naja atra*, both of which were collected from Zhejiang province, China. The protocol was approved by the ethics committee of Kunming Institute of Zoology, CAS, China.

### cDNA library construction and sequencing

Total RNA was extracted from venom glands using the RNeasy Mini Kit (Qiagen, Germany). The mRNA was purified from total RNA, using the Oligotex mRNA kit (Qiagen). The purified mRNA was used to make cDNA library, following the instructions in the SuperScript Plasmid System for cDNA Synthesis and Cloning Kit (plasmid used: pCMV-SPORT6 for *B. multicinctus*, pSPORT1 for *N. atra*) (Invitrogen, USA). Plasmids were purified using the QIAprep spin miniprep kit (Qiagen). Purified plasmids were sequenced by cycle sequencing reactions using the BigDye Terminator v3.1 kit (Applied Biosystem, USA) and an automated DNA sequencer (Model 3100A, Applied Biosystem, USA).

### cDNA sequence cluster assembly

After removing the vector, adaptors, and low-quality sequences, ESTs were assembled into contiguous clusters using the CAP3 program [45], with the setting that only joined sequences with at least 95% identity. Each cluster (contigs with more than one EST or singlets with one EST) was then searched against the GenBank databases using BLASTX and BLASTN algorithms to identify similar sequences with an e-value cutoff < 10^-5^, as described by Ching AT *et al*. [13]. The signal peptide was predicted using the local SignalP 3.0 server [46]. A final annotation table was generated in Microsoft Excel format, containing all the relevant information about clusters and singlets. EST sequences were deposited in GenBank dbEST under accession numbers HO056271 to HO058536.

### Bacterial artificial chromosome (BAC) library construction and characterization

High-molecular-weight DNA was extracted from snake blood cells, partially digested with *Eco*R I, and cloned into the CopyControl pCC1BAC vector (Epicentre, USA) according to a previously described method [47]. To estimate the recombination ratio of the libraries and the average size of inserted DNA fragments, 100 randomly selected BAC clone DNAs were digested with *Not *I and separated by PFGE, as described by Wang *et al*. [48].

### Screening the snake BAC genomic library

All the colonies in 384-well microtiter plates were replica-plated onto nylon membranes for hybridization using standard techniques [49]. Two rounds of screening were performed. In the first one, a mixture of 12 probes was used, which represented four kinds of toxin genes. These probes were respectively prepared for the consensus toxin coding sequences by labeling their PCR product with digoxigenin. They were designed based on the cDNA sequences of 3FTx, *β *bungarotoxin A-chain (or PLA2), *β *bungarotoxin B-chain, and NP (Additional file 2: Table S3). Then, the 79 positive clones were respectively hybridized with each kind of probe to exclude false positives and identify the concrete toxin gene type.

### Subcloning 3FTx genes and sequencing

All 39 3FTx gene-positive clones, verified by colony hybridization, were digested with *Eco*R I and analyzed on 0.8% agarose gels prior to blotting on a nylon membrane. Then, 3FTx gene-containing fragments were identified by hybridization with the same probes used in the library screening and subcloned into the CopyControl pCC1BAC vector. These subclones were then tested by PCR using specific primers, designed based on the sequence of the toxin ESTs (Additional file 2: Table S3). PCR amplifications were then ligated into the pMD18-T vector (TaKaRa, Dalian), and the fragments were sequenced using an ABI 3100A Genetic Analyzer. The sequences obtained from the same subclone were assembled into error-free consensus sequences using SeqMan II software (DNAStar, Version 6.0). These sequences were compared with our ESTs and published sequences in the GenBank database using the BLASTn program. Exon-intron structures of genes were double-checked using the Sim4 Program for spliced alignments [50].

### Evolutionary analyses

DNA or protein sequences were aligned with the MUSCLE program [51] with manual adjustments. The number of nucleotide substitutions per site (*Kn*) in the non-coding regions (introns) was estimated using the Kimura 2-parameter model, and the numbers of nucleotide substitutions per synonymous site (*dN*) and per nonsynonymous (*dS*) in the putative protein-coding regions were computed for pairs according to the method of the realistic evolutionary model in PAML [52]. Neighbor-joining (NJ) trees were constructed using the software MEGA version 4 [53], and the maximum likelihood (ML) trees were constructed using phylip-3.68 [54], evaluated by 1000 bootstrap replications, considering ML bootstrap proportions > 70% to indicate significant support, with transition-transversion ratios estimated from the data (implemented in tree-puzzle-5.2 [55]). There are several inframe indels in the coding region of ESTs, and those sites were removed in the sequence analysis.

In order to detect selection in a toxin subfamily, the likelihood ratio test (LRT), implemented in PAML4.2, was employed to detect positive selection using both the tests of M1a versus M2a and M8 versus M7 comparisons with the phylogeny-based approach [19,56]. The putative coding sequences of ESTs that showed high divergence from the other sequences in a sub-family were removed from the analysis.

## Authors' contributions

YL, XX, YZ, and RZ constructed the cDNA and BAC libraries. YJ and WL analyzed the data and wrote the manuscript. YZ and WW are the principal investigators who conceived the study and critically reviewed the manuscript. All authors read and approved the final manuscript.

## Supplementary Material

Additional file 1 **The complete list of all EST clusters in *B. multicinctus *and *N. atra*, annotated by using BLAST program**. Click here for file

Additional file 2 **Figure S1 and S2. Phylogenetic trees constructed, respectively, using Kunitz and lectins sequences with the maximum likelihood method in MEGA**. Figure S3. Characterization of the snake BAC libraries. Sixteen randomly selected *B. multicinctus *and *N. atra *BAC clones were digested with *Not *I and separated by PFGE. Lane M: Low-range PFG marker. Table S1. The representative sequences used for estimation of the dN/dS ratios. Table S2. The detailed information of all 71 positive toxin BAC clones from the toxin probe screening. Table S3. The toxin ESTs used as hybridization probes to screen for venom genes. Click here for file
