# Supplementary material for: Venom gland transcriptomes of two elapid snakes (Bungarus multicinctus and Naja atra) and evolution of toxin genes
Source: BMC Genomics. 2011 Jan 3;12:1. doi: 10.1186/1471-2164-12-1 (PMC3023746; doi:10.1186/1471-2164-12-1)
Supplement: Additional file 2 — Figure S1 and S2. Phylogenetic trees constructed, respectively, using Kunitz and lectins sequences with the maximum likelihood method in MEGA. Figure S3. Characterization of the snake BAC libraries. Sixteen randomly selected B. multicinctus and N. atra BAC clones were digested with Not I and separated by PFGE. Lane M: Low-range PFG marker. Table S1. The representative sequences used for estimation of the dN/dS ratios. Table S2. The detailed information of all 71 positive toxin BAC clones from the toxin probe screening. Table S3. The toxin ESTs used as hybridization probes to screen for venom genes. [file 1471-2164-12-1-S2.DOC]

**Figure S1**


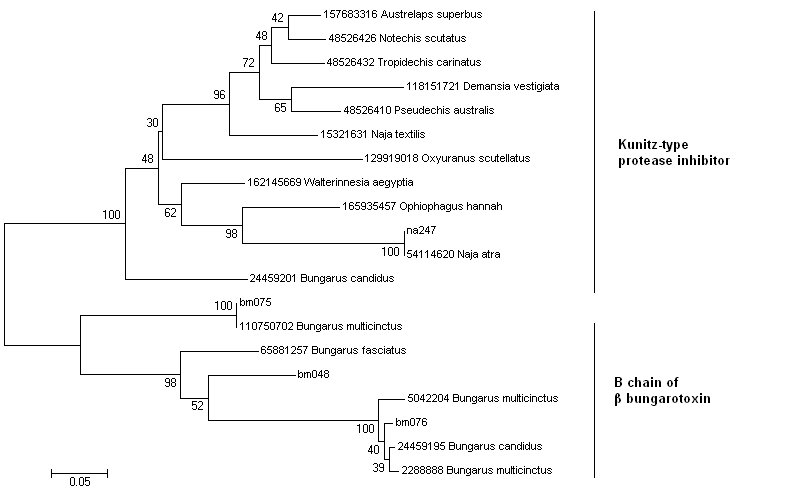


**Figure S2**

**
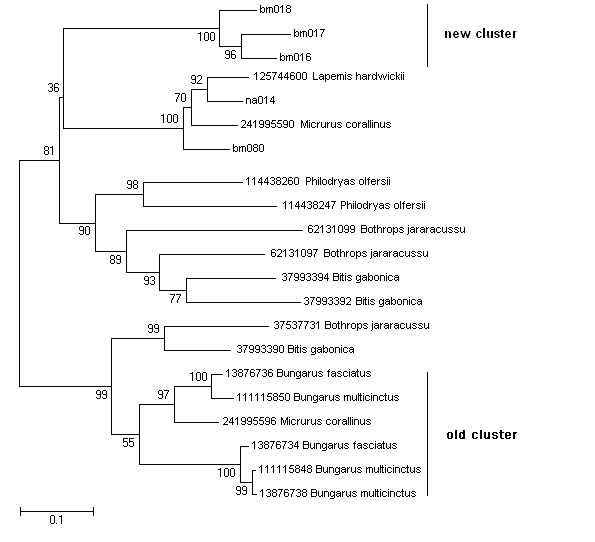
**

**Figure S3**

**
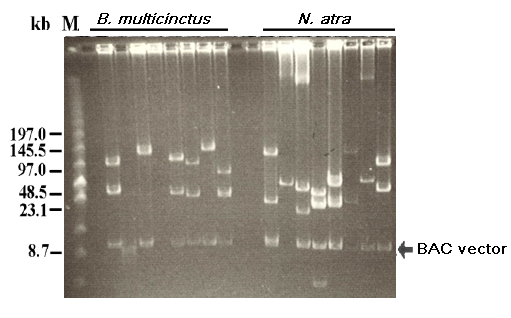
**

**Table S1**

| Gene  group | sequence  number | sequence GI ID  (tre file type) |
| --- | --- | --- |
| orphan II | 4 | (((na019,na003),na007),Bc47834353); |
| long chain | 4 | ((Ls3551477,Ns29465643),bm046,bm001); |
| short chain | 4 | ((na006,na005),na004,Ls4519807); |
| cytotoxin | 4 | ((na001,na054),na002,Oh82570092); |
| PLA2 (only) | 4 | ((Oh10863759,na022),Ls21734659,bm008); |
| PLA2 | 6 | ((Oh10863759,na022),Ls21734659,(bm008,(bm072,bm071))); |
| lectins | 7 | (((bm017,bm016),bm018),Co241995588,(Co241995590,(bm080,na014))); |
| Bbeta | 4 | ((bm048,Bf165881255),bm076,bm075); |
| Kunitz | 3 | (Wa162145671,Oh165935455,na247); |
| LAO | 5 | (((Bm126035648,Na126035676),Ns68304017),Pa68304019,Dv118151719); |
| Metal | 8 | ((((Om145982763,Ns145982765),As111572526),Dv118151737),(Nm21435682,Bm126035639),(Oh124007158,Na126035682)); |
| CRISP | 8 | ((((na061,Ls21305554),bm020),Oh225547743),(As158262801,Pp23264043),(na033,Oh28972960)); |
| NP | 6 | (((bm026np1,bm026np2),Da19848251),(Mc1684873,Mf71727514),Os66475093); |
| NGF | 9 | (((Bm266298,Tc76886036),(Tc76886038,Pa76886026)),(Dv118151729,(Ns46411220,(Om76886010,Nt76886014))),Ns46411222); |
| vespryn | 7 | (((((Dv118151739,Os158605248),Nt158605240),Ns158605242),Pa158605262),Pp158605260,Oh70907885); |

**Table S2**

| BAC name | Probe  kinds | **Lane NO. Figure 4** | Redundant BAC cluster | Sequenced subclone number | GenBank_Accn |
| --- | --- | --- | --- | --- | --- |
| N044_7B | 3FTx | 14 |  | 4 | HM597720, HM597721, HM597722, HM597723 |
| N076_20M | 3FTx | 10 |  | 3 | HM597732, HM597716, HM597718 |
| B132_1G | 3FTx | 3 |  | 2 | HM597741, HM597750 |
| B218_4L | 3FTx | 8 |  | 2 | HM597743, HM597744 |
| B241_M14 | 3FTx,NP | 35 | cluster 1 | 2 | HM597747, HM597748 |
| B244_1O | 3FTx | 19 | cluster 1 | 2 | HM597749, HM597737 |
| B254_9H | 3FTx | 6 | cluster 1 | 2 | HM597745, HM597746 |
| N031_7F | 3FTx | 33 |  | 2 | HM597725, HM597724 |
| N036_12B | 3FTx | 28 | cluster 4 | 2 | HM597726, HM597714 |
| N052_20J | 3FTx | 13 |  | 2 | HM597729, HM597719 |
| N061_6E | 3FTx | 21 |  | 2 | HM597731, HM597715 |
| N076_21M | 3FTx | 16 |  | 2 | HM597733, HM597734 |
| B214_13K | 3FTx | 20 |  | 1 | HM597753 |
| B229_14B | 3FTx | 9 | cluster 3 | 1 | HM597740 |
| B236_19K | 3FTx | 2 | cluster 1 | 1 | HM597752 |
| B250_20E | 3FTx | 4 |  | 1 | HM597751 |
| B296_10F | 3FTx | 7 |  | 1 | HM597736 |
| B307_7I | 3FTx | 24 | cluster 3 | 1 | HM597742 |
| B310_19J | 3FTx | 18 |  | 1 | HM597738 |
| B338_13B | 3FTx | 22 |  | 1 | HM597739 |
| N043_2E | 3FTx | 29 | cluster 4 | 1 | HM597727 |
| N044_7C | 3FTx | 31 |  | 1 | HM597728 |
| N059_9H | 3FTx | 15 |  | 1 | HM597730 |
| N076_1E | 3FTx | 11 |  | 1 | HM597717 |
| N103_16B | 3FTx | 12 |  | 1 | HM597735 |
| B322_12D | 3FTx | 5 | cluster 2 |  |  |
| B322_13E | 3FTx | 37 | cluster 2 |  |  |
| B296_12I | 3FTx | 26 | cluster 3 |  |  |
| N047_16J | 3FTx | 27 | cluster 4 |  |  |
| N052_10D | 3FTx | 32 |  |  |  |
| B144_7F | 3FTx | 1 |  |  |  |
| B165_1O | 3FTx | 17 |  |  |  |
| B196_19M | 3FTx | 23 |  |  |  |
| B294_8M | 3FTx | 25 |  |  |  |
| N046_5D | 3FTx | 30 |  |  |  |
| B230_10L | 3FTx | 34 |  |  |  |
| B228_16L | 3FTx | 36 |  |  |  |
| N159_19H | 3FTx,NP | 38 |  |  |  |
| B175_21N | 3FTx | 39 |  |  |  |
| B121_17O | Kuntiz |  |  |  |  |
| B137_17F | Kuntiz |  |  |  |  |
| B241_C7 | Kuntiz |  |  |  |  |
| B242_M144 | Kuntiz |  |  |  |  |
| B301_22P | Kuntiz |  |  |  |  |
| B312_17H | Kuntiz |  |  |  |  |
| B322_10F | Kuntiz |  |  |  |  |
| B358_17M | Kuntiz |  |  |  |  |
| B358_17O | Kuntiz |  |  |  |  |
| N038_18B | Kuntiz |  |  |  |  |
| N038_18C | Kuntiz |  |  |  |  |
| N102_18M | Kuntiz |  |  |  |  |
| N166_15O | Kuntiz |  |  |  |  |
| N169_20M | Kuntiz |  |  |  |  |
| B309_5N | NP |  |  |  |  |
| B323_17F | NP |  |  |  |  |
| B162_15E | PLA2 |  |  |  |  |
| B198_9C | PLA2 |  |  |  |  |
| B215_22L | PLA2 |  |  |  |  |
| B242_C7 | PLA2 |  |  |  |  |
| B242_D7 | PLA2 |  |  |  |  |
| B306_12F | PLA2 |  |  |  |  |
| B309_23M | PLA2 |  |  |  |  |
| B310_7L | PLA2 |  |  |  |  |
| B311_6N | PLA2 |  |  |  |  |
| B313_5H | PLA2 |  |  |  |  |
| B324_13M | PLA2 |  |  |  |  |
| B326_22N | PLA2 |  |  |  |  |
| B326_4F | PLA2 |  |  |  |  |
| B329_21C | PLA2 |  |  |  |  |
| B358_17P | PLA2 |  |  |  |  |
| B354_9K | PLA2,Kuntiz | |  |  |  |

**Table S3**

| probe category | GenBank_Accn | ESTs annotation | snake species |
| --- | --- | --- | --- |
| 3FTx | HO056831 | 3FTx long chain *α*-ntx | *B. multicinctus* |
| 3FTx | HO056891 | 3FTx kappa-neuro | *B. multicinctus* |
| 3FTx | HO056848 | 3FTx orphan group IV | *B. multicinctus* |
| 3FTx | HO057115 | 3FTx orphan group IXX | *B. multicinctus* |
| Kuntiz | HO056862 | *β* bungaratoxin B chain | *B. multicinctus* |
| NP | HO056785 | Natriuretic peptide | *B. multicinctus* |
| PLA2 | HO056870 | *β* bungaratoxin A chain | *B. multicinctus* |
| 3FTx | HO057622 | 3FTx short chain *α*-ntx | *N. atra* |
| 3FTx | HO057474 | 3FTx cytotoxin | *N. atra* |
| 3FTx | HO058164 | 3FTx orphan group I | *N. atra* |
| 3FTx | HO057799 | 3FTx orphan group II | *N. atra* |
| PLA2 | HO057808 | Phospholipase A2 | *N. atra* |
